# The silent threat: unruptured sinus of Valsalva aneurysm presenting as complete heart block

**DOI:** 10.1093/ehjimp/qyaf005

**Published:** 2025-01-23

**Authors:** Akshay Prashanth, Bharath Raj kidambi, Ramesh Sankaran, Nagendra Boopathy Senguttuvan, Periyasamy Thangavelu, Ranjith Karthikeyan, Vijay Kumar Muthayala

**Affiliations:** Department of Cardiology, Sri Ramachandra Institute of Higher Education and Research, No. 1, Sri Ramachandra Nagar, Mount Poonamallee Road, Porur, Chennai 600116, India; Department of Cardiology, Al-Dhannah Hospital, Ruwais, Abu Dhabi 11876, UAE; Department of Cardiology, Sri Ramachandra Institute of Higher Education and Research, No. 1, Sri Ramachandra Nagar, Mount Poonamallee Road, Porur, Chennai 600116, India; Department of Cardiology, Sri Ramachandra Institute of Higher Education and Research, No. 1, Sri Ramachandra Nagar, Mount Poonamallee Road, Porur, Chennai 600116, India; Department of Cardiothoracic Surgery, Sri Ramachandra Institute of Higher Education and Research, No. 1, Sri Ramachandra Nagar, Mount Poonamallee Road, Porur, Chennai 600116, India; Department of Cardiac Anesthesiology, Sri Ramachandra Institute of Higher Education and Research, No. 1, Sri Ramachandra Nagar, Mount Poonamallee Road, Porur, Chennai 600116, India; Department of Critical Care Medicine, Sri Ramachandra Institute of Higher Education and Research, No. 1, Sri Ramachandra Nagar, Mount Poonamallee Road, Porur, Chennai 600116, India

**Keywords:** unruptured sinus of Valsalva aneurysm, complete heart block, aneurysmectomy, chest pain

A 49-year-old male presented to the emergency department with sudden-onset dyspnoea, which had rapidly progressed to New York Heart Association Class IV over 1 week following recent chest trauma. An electrocardiogram (ECG) revealed a complete heart block with a ventricular escape rhythm (*Figure 1A*).

**Figure 1.**
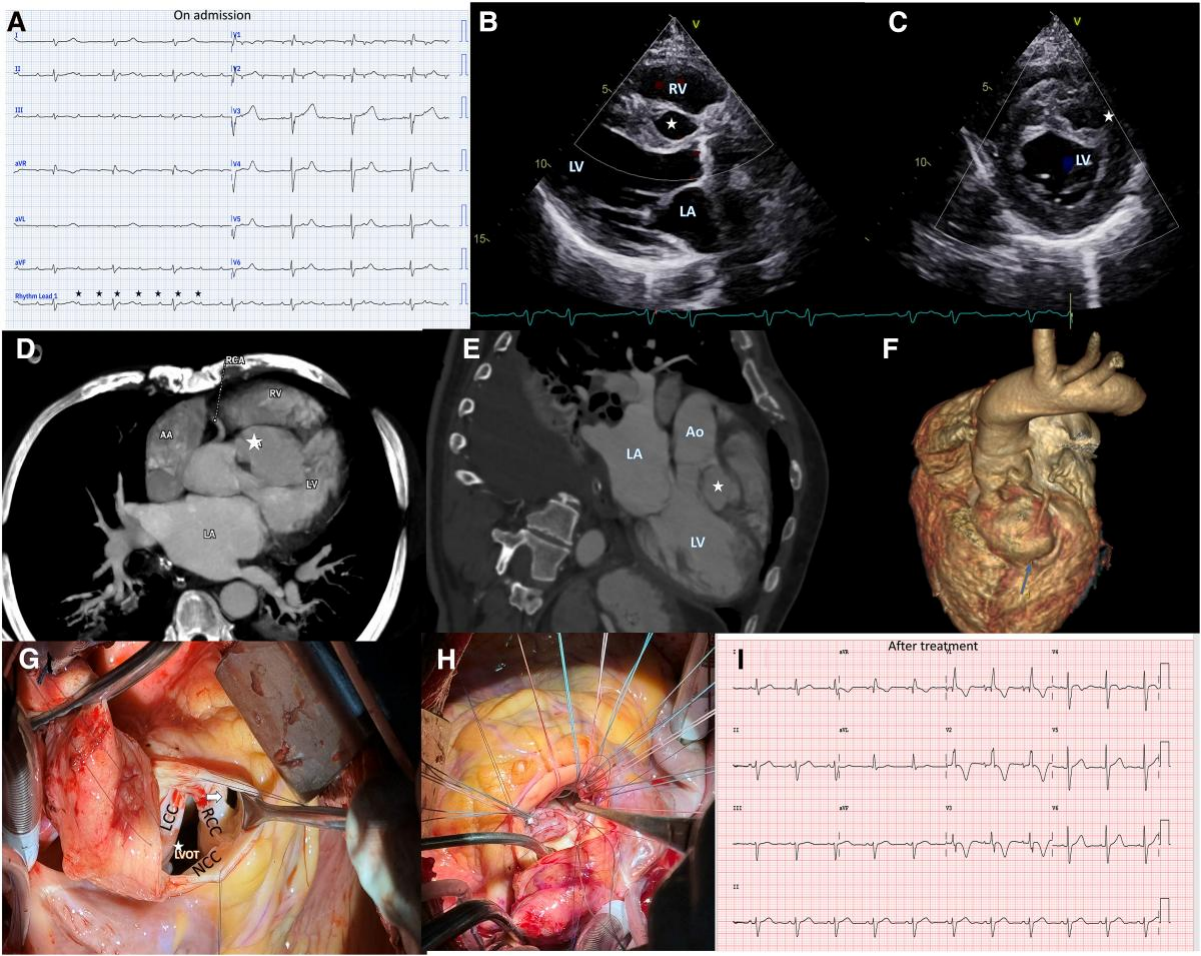
(*A*) Electrocardiogram at admission demonstrating complete heart block with a ventricular escape rhythm. (*B*, *C*) Transthoracic echocardiography (TTE) in the parasternal long-axis (*B*) and short axis (*C*) views, revealing an unruptured sinus of Valsalva aneurysm (white asterisk) protruding into the basal interventricular septum, causing significant compression of the conduction system. (*D*, *E*) Contrast-enhanced cardiac computed tomography (CT) images in axial (*D*) and LVOT (*E*) planes confirming a 5.1 × 3.3 cm aneurysm (asterisk) originating from the right sinus of Valsalva, extending into the interventricular septum without coronary artery compression. (*F*) Three-dimensional volume-rendered CT reconstruction visualizing the aneurysm's anatomical relationship with adjacent cardiac structures. (*G*, *H*) Intraoperative images showing the aneurysm repair. (*G*) Exposure of the aneurysm sac during surgery. (*H*) Surgical closure using an autologous pericardial patch along with aortic valve replacement. (*I*) Postoperative electrocardiogram demonstrating resolution of the complete heart block, with restoration of sinus rhythm and a right bundle branch block. RCC (right coronary cusp), NCC (non-coronary cusp), Ao (aorta), RV (right ventricle), LV (left ventricle), and LA (left atrium).

Transthoracic echocardiography and transoesophageal echocardiography showed an unruptured sinus of Valsalva aneurysm protruding into the basal inter-ventricular septum, causing significant compression of the conduction system (*Figure 1B* and *C*; see Supplementary data online, *Video S1*). Cardiac computed tomography confirmed a 5.1 × 3.3-cm aneurysm arising from the right sinus of Valsalva, extending into the inter-ventricular septum without compressing the coronary arteries (*Figure 1D* and *E*).

The patient underwent successful surgical closure using an autologous pericardial patch along with aortic valve replacement (*Figure 1G* and *H*). The post-operative course was uneventful, and follow-up ECG demonstrated resolution of the heart block with normalization of sinus rhythm and right bundle branch block (*Figure 1I*). Follow-up imaging confirmed no residual aneurysm and no aortic insufficiency.

This case highlights a rare presentation of an unruptured sinus of Valsalva aneurysm causing complete heart block by compressing the conduction system. Early detection and surgical intervention are essential to prevent catastrophic complications such as rupture or progressive conduction disturbances.

## Supplementary Material

qyaf005_Supplementary_Data

## Supplementary data

**Consent:** Patient consent obtained and no patient identifiable information present in the manuscript to the best of our knowledge.

**Funding:** None declared.

**Data availability:** No new data were generated or analysed supporting this research.

## Lead author biography

S. Ramesh, MD, DM (Cardiology), is a Professor and Head of the Department of Cardiology at Sri Ramachandra Institute of Higher Education and Research, Chennai. With over 15 years of experience, he has led research in genotype-guided antiplatelet therapy and coronary artery disease. Dr Ramesh has published extensively on CYP2C19 polymorphisms in acute coronary syndrome and interventional cardiology. A Fellow of the American College of Cardiology (FACC), European Society of Cardiology (FESC), and FSCAI, he is highly regarded for his contributions to both clinical practice and cardiovascular research.

